# Microcystin Levels in Selected Cyanobacteria Exposed to Varying Salinity

**DOI:** 10.4236/jwarp.2019.114023

**Published:** 2019-04

**Authors:** Dy’mon Walker, Somayeh Gharaie Fathabad, Behnam Tabatabai, Sanjeeda Jafar, Viji Sitther

**Affiliations:** 1 Department of Biology, Morgan State University, Baltimore, MD, USA; 2 Medical Technology Program, Morgan State University, Baltimore, MD, USA

**Keywords:** Cyanotoxin, ELISA, Harmful Algal Blooms, Protein Expression, Sodium Chloride

## Abstract

Microcystins produced by cyanobacteria pose a great threat to human health by releasing toxins upon cell death. In the present study, we studied microcystin production in the cyanobacterial strains *Anabaena cylindrica* (B629 and 2949) and *Fremyella diplosiphon* (SF33) exposed to 1, 2 and 4 g/L sodium chloride (NaCl). Cultures grown for 7 days in BG11/HEPES medium were pelleted, re-grown in the corresponding NaCl levels, and enzyme linked immunosorbent assay (ELISA) performed. ELISA assays revealed enhanced microcystin production in *A. cylindrica* B629 exposed to 4 g/L NaCl and *A. cylindrica* 29414 exposed to 2 and 4 g/L NaCl, after growth in the corresponding NaCl levels for 14 days. We observed a significant decrease (p >0.05) in microcystin levels in the control strains after exposure to NaCl for 5 days. After exposure to 1, 2, or 4 g/L NaCl for 10 days, no microcystin release was observed in *A. cylindrica* B629, *A. cylindrica* 29414 or F. *diplosiphon* SF33. Sodium dodecyl sulfate polyacrylamide gel electrophoresis identified the presence of an additional band at 120 – 130 kDa in *A. cylindrica* B629 exposed to 2 and 4 g/L NaCl, and at 14 kDa in cultures amended with 1 and 2 g/L NaCl as well as the untreated control, indicating that exposure to salinity induces alterations in protein expression.

## 1. Introduction

Massive proliferation of cyanobacterial blooms has severe ecological impacts including changes in biodiversity and depletion of available oxygen resulting from eutrophication [1]. Certain cyanobacterial species that are known to form harmful algal blooms (HABs) produce toxins impacting human health and aquatic biota. The intracellular cyanotoxins released by the rupture and disintegration of cyanobacterial cell membrane during cell lysis trigger these toxins to become extra-cellular and persist in marine environments for several months [2] [3]. Micro-cystins, the most significant cyanotoxins, are known to inhibit liver function, disrupt sperm motility and morphology, cause intestinal inflammation, impair spatial memory, and trigger neuronal degenerative changes and apoptosis in vertebrates [4] [5] [6] [7]. At the molecular level, microcystins also affect humans by binding to okadaic acid receptors, which promote tumor formation and inhibit serine/threonine protein phosphatases 1 (PP1) and 2A (PP2A) [8].

Various abiotic factors such as light and temperature influence microcystin concentration by influencing the abundance of microcystin-producing strains as well as by regulating extracellular and intracellular abundance and toxicity [9]. In addition, nitrogen, phosphorus, and HAB-causing chemicals from agricultural runoff are reported to increase microcystin production [10]. Of these factors, salinity has a significant impact on water quality and aquatic life due to the enormous amounts of de-icing salts released into water bodies. Studies have shown that salt from the run-off is washed downstream, thus increasing the salinity of streams and lakes [11] [12].

While microcystin production in various cyanobacterial species has been reported [13] [14], there is no comparative study of microcystins produced among the cyanobacteria, *Anabaena cylindrica* B629 and 29414 (known to produce microcystin) and *Fremyella diplosiphon* SF33 (not reported to produce microcystin). In this study, the impact of 1, 2, and 4 g/L sodium chloride (NaCl) on total microcystin production in these strains was studied. In addition, we identified changes in protein expression in these strains exposed to 1, 2 and 4 g/L NaCl using sodium dodecyl sulfate polyacrylamide gel electrophoresis (SDS-PAGE).

## 2. Materials and Methods

### 2.1. Strains and Culture Conditions

*A. cylindrica* B629 was obtained from the UTEX culture collection, *A. cylindrica* 29414 from the ATCC collection, and *F. diplosiphon* SF33 strain from Dr. Montgomery’s laboratory (Michigan State University).Cultures were grown on BG-11 solid medium containing 20 mM HEPES at 28°C for 5 – 7 days under continuous white light adjusted to 30 μmol·m^−2^s^−1^ using a LI-190SA quantum sensor (Li-Cor, USA). Cells were transferred to 125 mL Erlenmeyer flasks containing 50 mL liquid BG11/HEPES at an initial optical density of 0.1 at 750 nm (OD_750_). Cultures were grown under continuous shaking at 170 rpm at 28°C. Three replicate treatments were maintained and the experiment repeated once.

### 2.2. Culture Preparation and Enzyme-Linked Immunosorbent Assay (ELISA)

Cyanobacterial cultures were grown in salt free BG11/HEPES medium for 7 days. At day 7, cells were centrifuged at 6000 rpm for 4 min, supernatant discarded, and the pellet transferred to BG11/HEPES supplemented with 1, 2, or 4 g/L NaCl. Three replicates were maintained for each strain tested. Cultures grown in the absence of NaCl served as control. On day 14, cells were disrupted using a sonicator (Fisher Scientific, CL18) at 100% amplification for 90 s and stored at −20°C overnight.

To determine the microcystin levels, ELISA was performed using Microcystin-ADDA ELISA test kit (ABRAXIS, Inc.). In this method, 50 μL of the standard, control, or treatment was added to the test-strip wells followed by 50 μL antibody solution. Wells were covered with parafilm, contents mixed in a rocking shaker for 30 seconds, followed by incubation at room temperature for 90 minutes. Strips were washed three times by 1× wash buffer solution, decanted, and plate inverted for roughly one minute. Then, 100 μL enzyme conjugate solution was added to each well, covered with parafilm, mixed in a shaker for 30 seconds, and incubated at room temperature for 30 minutes. Wells were washed with the same buffer thrice, decanted, and inverted for about one minute. Then, 100 μL of substrate (color) solution was added to individual wells followed by mixing for 30 seconds, and incubated at room temperature for 30 minutes. Finally, 50 μL of stop solution was added and absorbance was read at 450 nm using a microplate ELISA photometer within 15 minutes. Microcystin concentrations were determined by comparison to the supplied calibrators and the experiment repeated once [14].

### 2.3. Microcystin Production over Time

*A. cylindrica* B629 and 29414 and F. *diplosiphon* SF33 strains were grown in BG-11/HEPES under conditions mentioned above for a period of 10 days and 1 mL aliquots obtained from each strain at 5-day intervals (Days 0, 5, 10). Cells were sonicated at 100% amplification for 90 s and stored at −20°C overnight. ELISA assays were performed the following day as mentioned above.

### 2.4. Comparison of Protein Expression in Cyanobacterial Cultures Exposed to Varying NaCl Levels Using 1D-PAGE

**Figure 1.**
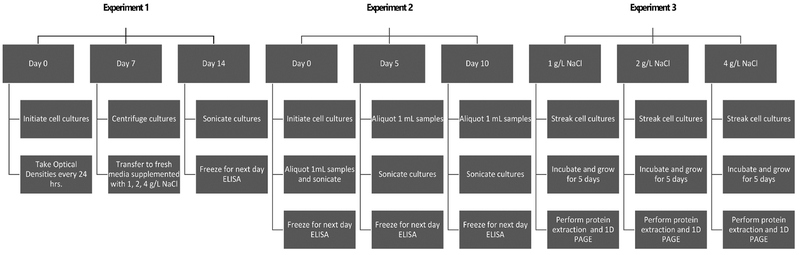
Summarized timeline of experiments followed in the present study.

Total protein was extracted according to the protocol described by Kendrick Labs (USA). Protein isolation was performed using osmotic lysis buffer (10 mM Tris, pH 7.4, and 0.3% SDS) containing 10× nuclease stock, 100× phosphatase inhibitor stocks I & II, and 100× protease inhibitor stock according to the manufacturer’s protocol. Five-day old *A. cylindrica* B629 and 29414 and *F. diplosiphon* SF33 cultures were grown on liquid BG-11/HEPES supplemented with 1, 2, and 4 g/L NaCl as mentioned above. Cells grown in the absence of NaCl served as control. Approximately 50 – 75 mg of the control and NaCl-treated cells were collected, vortexed, and lysed by inversion in 500 μl of the osmotic lysis buffer master mix followed by 30 s sonication at 100% power. Aliquots of 25 μl were removed from each sample and protein concentration determined using the Pierce Bicinchoninic acid (BCA) Assay (Thermo Fisher Scientific, USA). SDS boiling buffer without BME (5% SDS, 10% glycerol and 60 mM Tris, pH 6.8) (500 μl) was then added to samples and heated in a water bath at 98°C for 5 min. Samples were lyophilized and re-dissolved to 4 mg/ml in 1:1 mixture of diluted SDS boiling buffer (5% SDS, 5% BME, 10% glycerol and 60 mM Tris, pH 6.8) and urea sample buffer (9.5 M urea, 2% w/v IGEPAL CA-630, 5% BME). Aliquots (50 mg) of each sample were run on 10% polyacrylamide gel in a MiniProtean Tetra Gel system at 150 V for 90 min. The gel was washed three times (5 min each rinse) with distilled water and protein bands visualized using Simply Blue stain. A comprehensive work flow of steps followed in this study is provided in Figure 1

## 3. Results

### 3.1. Microcystin Quantification in *A. cylindrica* B629 and 29414 and *F. diplosiphon* SF33

**Figure 2.**
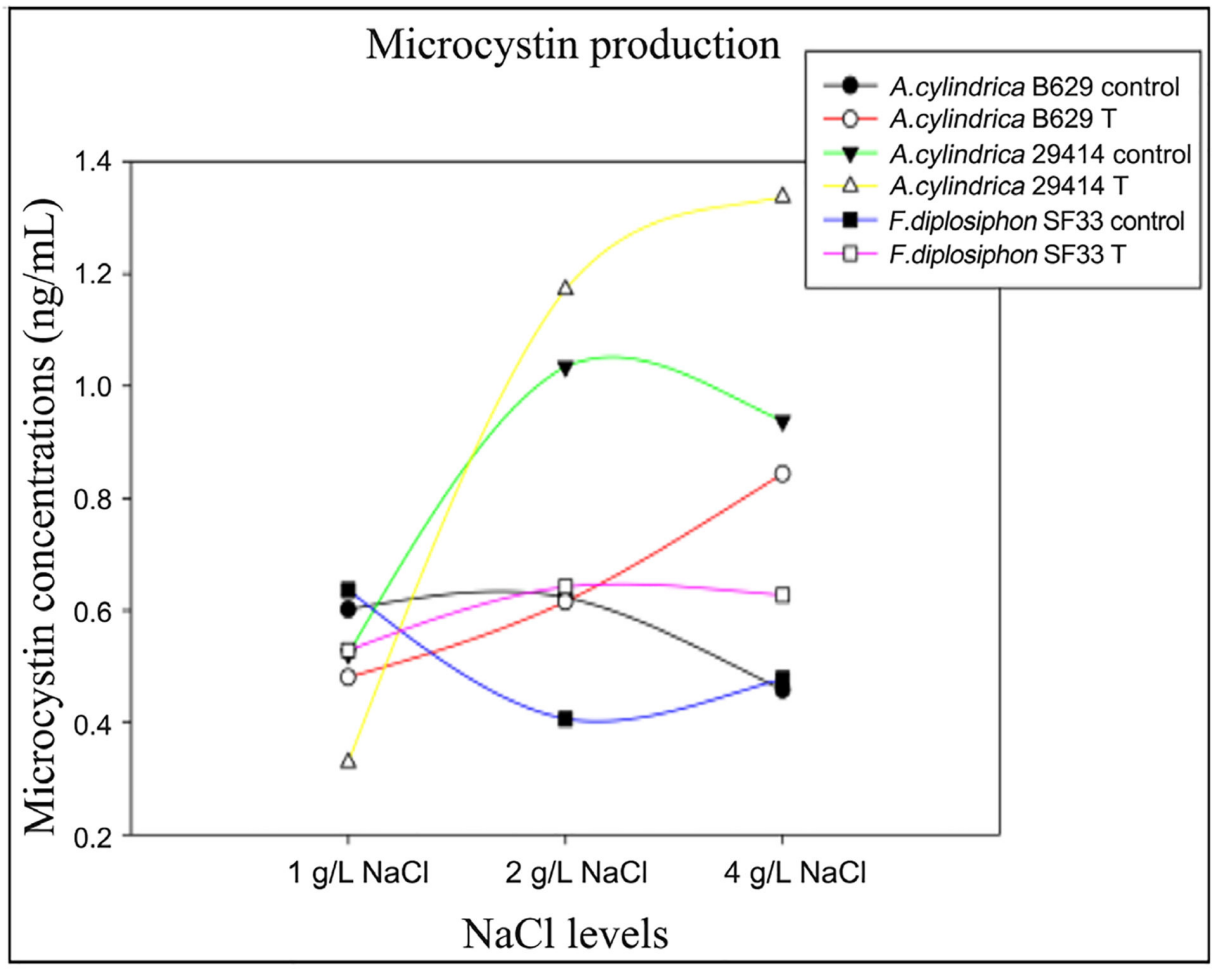
Microcystin production in *Anabaena cylindrica* B629 and 29414 and *Fremyella diplosiphon* SF33 cultures exposed to 1 g/L, 2 g/L, and 4 g/L sodium chloride (NaCl) and untreated control.

We observed a significant increase (p < 0.05) in microcystin levels of *A. cylindrica* B629 and *A. cylindrica* 29414 strains exposed to 4 g/L NaCl compared to their corresponding control (Figure 2, Table 1(a)). In addition, we observed a significant increase (p < 0.05) in microcystin levels of *A. cylindrica* 29414 exposed to 4 g/L NaCl compared to *F. diplosiphon* SF33.

### 3.2. Effect of NaCl on Microcystin Production in *A. cylindrica* B629 and 29414 and *F. diplosiphon* SF33 over a Period of 10 Days

**Figure 3.**
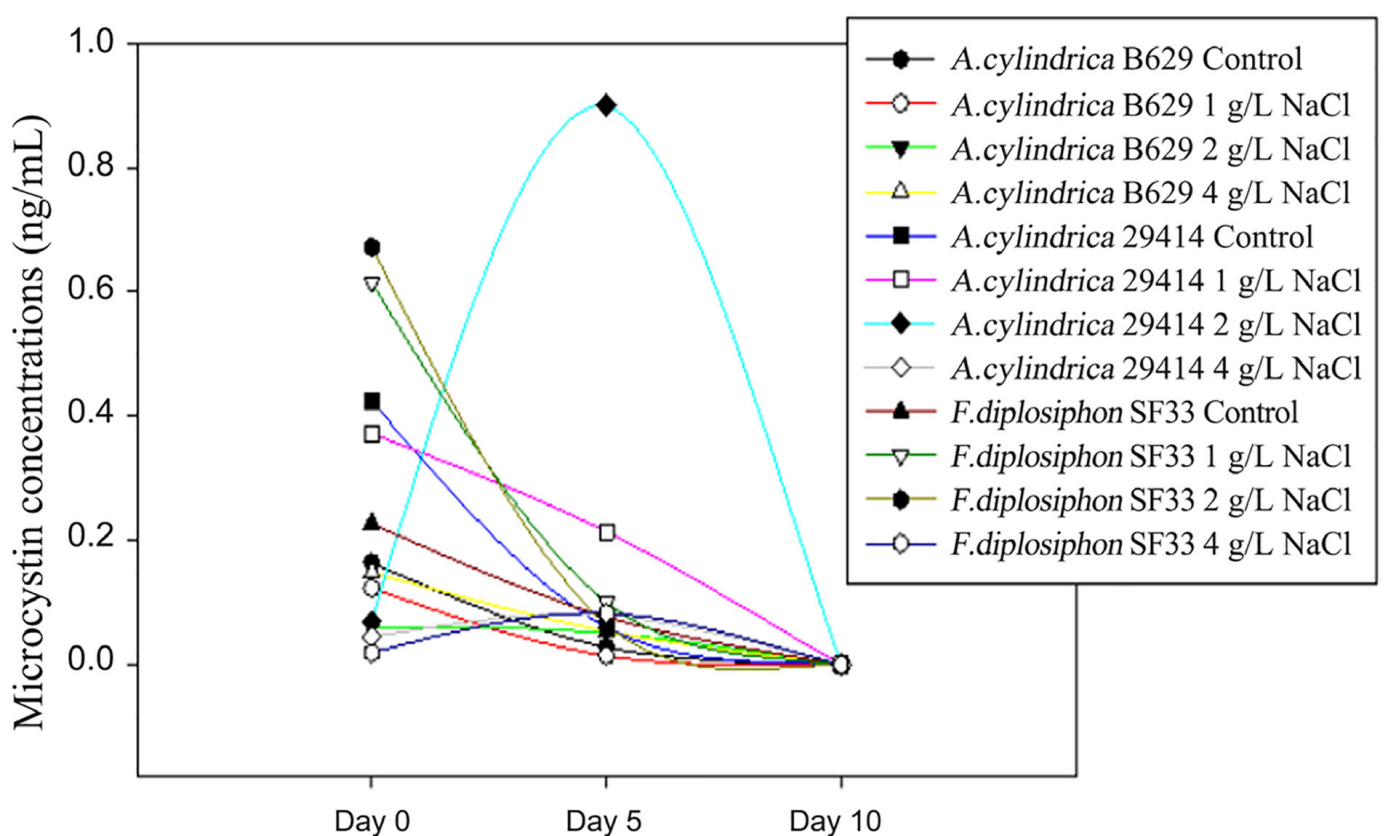
Microcystin production in *Anabaena cylindrica* B629 and 29414 and *Fremyella diplosiphon* SF33 cultures exposed to sodium chloride (NaCl) over a period of 0, 5, and 10 days.

We monitored microcystin production for a period of 10 days to identify changes in microcystin release over time. Our results revealed no significant differences (p > 0.05) in microcystin levels at day 0 in F. *diplosiphon* SF33, *A. cylindrica* B629 and 29414 strains grown in salt-free medium (Figure 3; Table 1(a)). In addition, we observed a significant decrease (p > 0.05) in microcystin levels of *A. cylindrica* B629 and *F. diplosiphon* SF33 grown in salt-free medium after five days compared to their initial levels. At day 10, no microcystin was detected in all the three strains exposed to 1, 2, and 4 g/L NaCl.

### 3.3. Protein Expression in *A. cylindrica* B629 and 29414, and *F. diplosiphon* SF33 Cultures Exposed to NaCl

**Figure 4.**
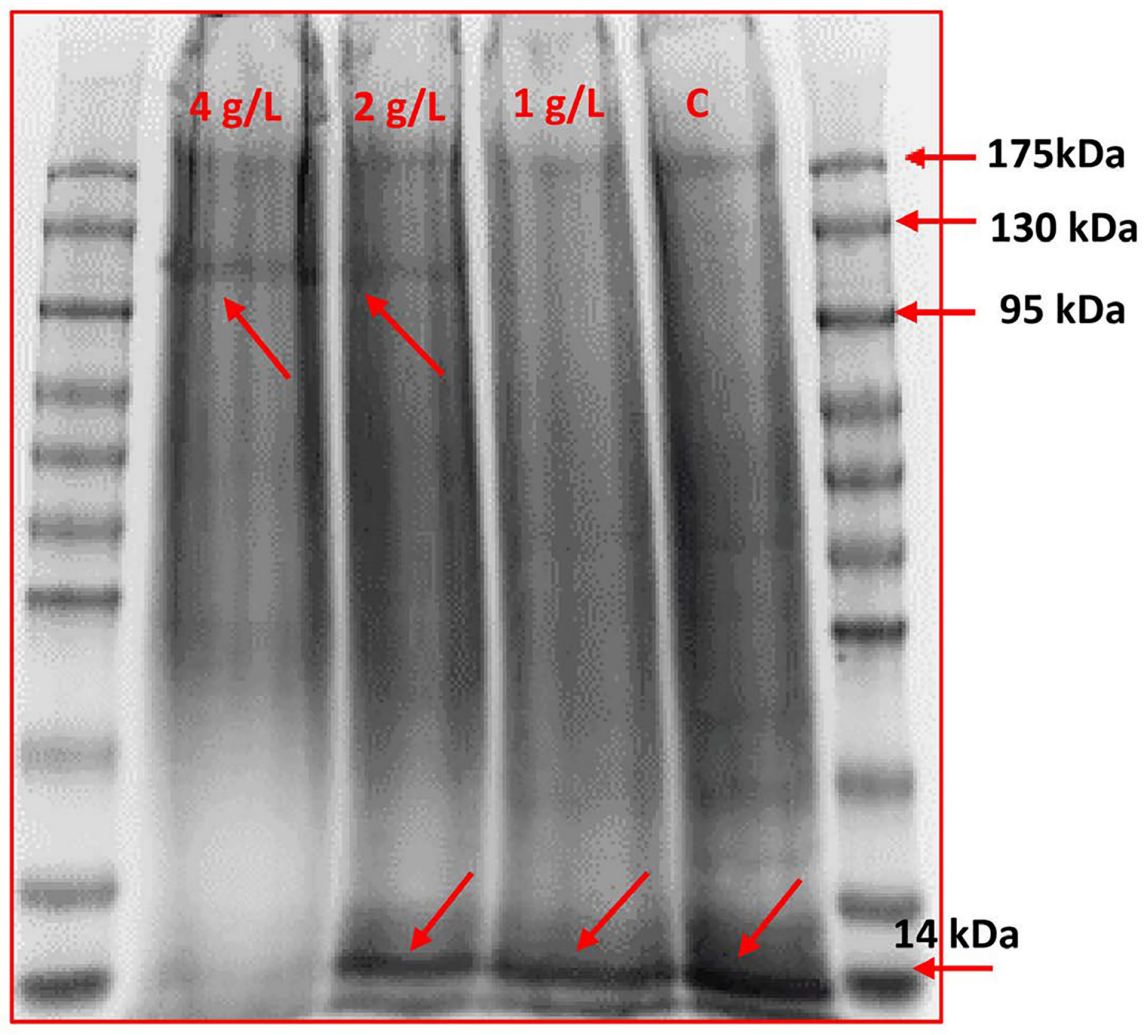
Sodium dodecyl sulfate polyacrylamide gel electrophoresis (SDS-PAGE) of *Anabaena cylindrica* B629 treated with 1, 2, and 4 g/L sodium chloride (NaCl) and untreated control (C).

SDS-PAGE revealed differential protein expression in cultures exposed to 1, 2, and 4 g/L NaCl over a period of 10 days. A single band at 120 – 130 kDa was visualized only in cultures treated with 2 g/L and 4 g/L NaCl (Figure 4). A 14 kDa band was observed in control *A. cylindrica* cultures as well as those exposed to 1 and 2 g/L NaCl. Additionally, our results revealed the presence of a band at 175 kDa in all *A. cylindrica* B629 cultures. By contrast, the band at 120 – 130 kDa was not observed in *A. cylindrica* 29414 and *F. diplosiphon* SF33.

## 4. Discussion

Increasing frequency and intensity of HABs pose serious environmental concerns resulting in high mortality rates of invertebrate and vertebrates and plant populations [11]. HABs are caused by nutrient overload or chemical pollutants commonly found in products used in agriculture [13]. A number of cyanobacterial species including *Microcystis, Anabaena*, *Oscillatoria, Nodularia, Nostoc, Gloeotrichia, Anabaenopsis,* and terrestrial *Hapalosiphon* sp. have been recognized to produce microcystin [3] [12] [14].

Several studies have reported the impact of environmental factors such as light and temperature on the prevalence of HABs [9]. In addition, various nutrients such as nitrogen and phosphorus have been reported to affect cyanotoxin release [15]. However, a study on the impact of varying salinity exposure on microcystin production and protein expression in *A. cylindrica* sp. and *F. diplosiphon* has not been clearly elucidated. Our findings indicate increased microcystin levels in *A. cylindrica* B629 cultures exposed to 4 g/L NaCl, and *A. cylindrica* 29414 supplemented with 2 and 4 g/L NaCl (Figure 2), suggesting that higherNaCl levels could lead to enhanced microcystin release. These results are in accordance with those of Engström-Öst *et al.* [16] and Tonk *et al.* [17], who reported that salinity influences extracellular microcystin production in *Anabaena* and other microcystin-producing species.

Further, results of our study revealed a correlation in microcystins produced by these strains over a period of 10 days in response to salt shock. At day 5, microcystin level decreased in *A. cylindrica* B629 relative to day 0 (Figure 3), suggesting that prolonged exposure to NaCl could have led to the degradation of microcystins. These findings suggest that microcystin levels vary during the cell growth cycle in the *A. cylindrica* strains. No microcystin was detected at day 10 in all three strains exposed to the varying NaCl concentrations tested. In a similar study by Tonk *et al.* [17], microcystin concentrations produced by *Microcystis aeruginosa* were found to increase at salinity levels above 10 g/L.

The presence of an additional band at 120 – 130 kDa in *A. cylindrica* B629 cultures exposed to 2 and 4 g/L NaCl, and a band at 14 kDa in cultures exposed to 0, 1 and 2 g/L NaCl (Figure 4) suggests that elevated levels of NaCl could result in alterations at the protein level. Since the 14 kDa microcystin-associated protein histidine phosphatase dephosphorylates ATP-citrate lyase [18], presence of the band at this specific size for *A. cylindrica* B629 control and cultures exposed to 1 and 2 g/L NaCl suggests ATP dephosphorylating properties, however high salinity levels may deactivate these mechanisms.

## 5. Conclusion

In summary, our results indicate that exposure of these cyanobacterial strains to NaCl could induce microcystin production and expression of ATP-citrate lyase dephosphorylating proteins. While lower salinity levels were tested in this study, it is possible that exposure to elevated NaCl levels will offer a more comprehensive insight on the effect of NaCl on microcystin production and release. Future studies will also aim to investigate alterations in protein expression in these strains using matrix-assisted laser desorption/ionization time-of-flight/time-of-flight mass spectrometry to identify specific microcystin-associated proteins. These studies will enhance our understanding of microcystins on cellular and metabolic processes, and provide pathways to mitigate their detrimental effects on the hydrosphere.

## Figures and Tables

**Table 1.** (a) Significance values for studies on microcystin quantification in *Anabaena cylindrica* B629 and 29414 and *Fremyella diplosiphon* SF33 and (b) Effect of sodium chlo-ride (NaCl) on microcystin production over a period of 10 days.

(a)		
Treatment pairs	p-value	Inference
B629 vs 29414 controls	0.0492958	*p < 0.05
29414 vs SF33 4 g/L NaCl	0.0294261	*p < 0.05
29414 1 g/L NaCl vs 29414 2 g/L NaCl	0.0337434	*p < 0.05
29414 1 g/L NaCl vs 29414 4 g/L NaCl	0.0157504	*p < 0.05
(b)		
Treatment pairs	p-value	Inference
B629 cultures after day 5	0.0061444	**p < 0.01
SF33 cultures after day 5	0.0363168	*p < 0.05
